# Type 1 Choroidal Neovascularization Evolution by Optical Coherence Tomography Angiography: Long-Term Follow-Up

**DOI:** 10.1155/2020/4501395

**Published:** 2020-04-24

**Authors:** Marco Rispoli, Maria Cristina Savastano, Bruno Lumbroso, Lisa Toto, Luca Di Antonio

**Affiliations:** ^1^Centro Italiano Macula, Rome, Italy; ^2^UOC Oftalmologia, Fondazione Policlinico Universitario A. Gemelli IRCCS, Rome, Italy; ^3^Università Cattolica del Sacro Cuore, Rome, Italy; ^4^Ophthalmology Clinic, National High-Tech Center in Ophthalmology, Italian School of Robotics in Ophthalmology, University “G. d'Annunzio” of Chieti-Pescara, Chieti, Italy

## Abstract

**Purpose:**

To evaluate structural changes in response to antivascular endothelial growth factor (anti-VEGF) treatment in patients with long-term type 1 choroidal neovascularization (CNV) by optical coherence tomography (OCT) and OCT angiography (OCTA).

**Method:**

This is a longitudinal study that involved a total of 51 eyes with type 1 CNV (35 female and 16 male eyes). Structural OCT and OCTA were performed on all the subjects. AngioVue OCTA (XR Avanti, Optovue, Inc., Fremont, CA) was used to obtain qualitative and quantitative information. All eyes were treated with an anti-VEGF ProReNata (PRN) approach and were followed for a mean of 38.9 months (SD ± 7.22). Best-corrected visual acuity (BCVA) was assessed at each follow-up timepoint.

**Results:**

We observed two kinds of possible evolution of type 1 CNV: “positive evolution,” including stabilization in 20% of patients and chronicity in 35%, and “negative evolution,” in which fibrosis was shown in 18% of patients, chorioretinal atrophy in 25%, and hemorrhage or RPE tears in 2%. The mean BCVA at baseline was 33.67 ± 15.85 ETDRS letters; after 1 and 2 years, it was 31.61 ± 18.04 and 31.18 ± 18.58 ETDRS letters, respectively. The mean BCVA at the end of follow-up was 25.27 ± 20 ETDRS letters. The difference between the values at baseline and at the end of follow-up was not statistically significant (*P* = 0.06, *r*^2^ = 0.10).

**Conclusions:**

This study describes an in vivo structural long-term evolution of type 1 CNV by OCT and OCTA. Different possible CNV outcomes were observed. This study suggests that new retinal imaging techniques could be useful tools for assessing the potential retinal changes in the evolution of type 1 CNV to develop personalized medicine. Further studies using OCTA in the long term are needed to better understand why similarly treated type 1 CNV cases evolve differently and produce different results.

## 1. Introduction

Noninvasive dyeless optical coherence tomography angiography (OCTA) is a clinical technique that is spreading rapidly all over the world, as it is safer, easier, and faster than fluorescein angiography (FA) and indocyanine green angiography (ICG) [1, 2]. Structural OCT highlights alterations in the morphology and structure of the retinal layers. OCTA provides images of blood flow in the retina and choroid with a high level of detail. In contrast, FA cannot show the vascular layers of blood vessels as deep as the capillary plexuses, which are well evidenced by OCTA [3]. This allows for several potential options of disease analysis, the research of different disorders, and the evaluation of new treatments [4].

One of the first pathologies studied by OCTA was wet AMD. The dyeless visualization of new vessels was remarkable for a large number of researchers around the world. OCTA enables the understanding, quantification, and tracking of the evolution after new vessel (NV) treatment.

CNV treatment should begin early, shortly after symptoms appear and before the occurrence of extensive structural damage. In the absence of a recognized guideline for the treatment and evaluation of the timing of eyes with exudative neovascularization, patients should be closely monitored for treatment and retreatment. Antivascular endothelial growth factor (anti-VEGF) treatment is universally recognized as providing positive results in the reduction of CNV activity and in maintaining good vision for patients for years [5–7].

Several trials (ANCHOR, MARINA, VIEW 1, and VIEW 2) have demonstrated visual improvement of approximately 10 letters at 2 years in eyes with neovascular AMD undergoing monthly anti-VEGF therapy [8–10]. Recently, 5-year results from the Comparison of Age-Related Macular Degeneration Treatment Trial (CATT) study showed long-term visual deterioration with chronic anti-VEGF therapy [11].

Although several factors may play a role in causing vision loss in eyes that undergo long-term anti-VEGF therapy, the mechanism of this process remains poorly understood. One possible reason has been postulated by Dansingani and Freund, in which a mature tangled vascular pattern in type 1 lesions was determined to be a resistance factor to macular atrophy [12].

Recently, Christenbury et al. described a high level of macular atrophy development predominantly eccentric to the PED in long-term anti-VEGF therapy for eyes with type 1 NV secondary to AMD [13]. Despite studies reporting results of chorioretinal atrophy and a decrease in BCVA, several other studies have reported a maintained or increased BCVA and OCT morphology improvement [14, 15].

The potential chorioretinal involvement after anti-VEGF treatment led us to investigate the evolution of type 1 CNV in exudative AMD eyes, which we analyzed with structural OCT and OCTA in a long-term follow-up study.

This research project is aimed at studying the particular CNV morphological changes seen on OCTA at the end of the observational period.

## 2. Methods

This study adhered to the Declaration of Helsinki (52^nd^ WMA General Assembly, Edinburgh, Scotland, October 2000), and written informed consent to participate in this study was routinely obtained from all examined patients. The IRB/ethics committee ruled that ethical approval was not required. In this cross-sectional study, fifty-one wet AMD eyes with type 1 CNV (35 female and 16 male eyes) detected by structural OCT according to a previous study [16] were evaluated. The mean age of the patients was 77.41 years, with a standard deviation (SD) of 12.39 years. All eyes were treated by an anti-VEGF ProReNata (PRN) approach and had follow-up every month. The duration of time followed for the entire cohort ranged from 31 months to 58 months, with a mean of 38.9 months (SD 7.22). The pharmacological agents ranibizumab and aflibercept were randomly chosen for administration. The number of intravitreal injections per eye during the course of treatment ranged from 3 to 29 injections, with a mean of 11.86 injections (SD 6.64). The patient demographics are listed in Table 1.

The exclusion criteria included media opacity and concomitant diseases such as diabetic retinopathy, vein or artery occlusion, glaucoma, any evidence or suspicion of type 2 and/or type 3 CNV, polypoidal choroidal vasculopathy, and any history of photodynamic therapy or macular laser therapy. Patients who presented with cataracts were followed without surgery because they did not have a clinically significant increase over time. All patients underwent a baseline ophthalmic examination, including medical and ocular history, family medical history, measurement of best-corrected visual acuity (BCVA) expressed in Early Treatment of Diabetic Retinopathy Study (ETDRS) letters, slit-lamp examination of the anterior and posterior segments, measurement of intraocular pressure, and dilated fundus examination. All eyes were imaged with an AngioVue OCTA (XR Avanti, Optovue, Inc., Fremont, CA) as the collected CNV assessment. The structural OCT protocol pattern used centered the B-scan line and crossline onto the fovea. The OCTA protocol used centered 3 × 3 mm^2^ and 6 × 6 mm^2^ grids onto the fovea. OCTA software programs automatically analyze retinal layer scans at different depths, providing images that are rich in details. With OCTA technology, the same tissue area is imaged repeatedly, and the differences between the scans are analyzed, thus allowing one to detect zones with high-flow neovascular rates. In cases of segmentation errors, manual editing of the layers was performed if deemed necessary for a correct interpretation. We classified all CNVs as inactive using biomarkers of CNV activity described by Al-Sheikh et al. [17] The OCTA scans that better represented the CNV features were selected and considered for analysis. We have chosen the images that agreed between CNV features and greater flows in B-scan. If the high flows were observed in the outer retina, the OCTA images in the outer retina were chosen. In case of main flows shown in the sub-RPE area, the OCTA scans were selected at this level. All images were analyzed by two of the authors (B.L. and M.C.S.) on two separate occasions to ensure accuracy of the grading. In cases of disagreement, both readers reanalyzed the images, and a consensus was obtained. The OCTA images analyzed were taken at the last visit. For statistical analysis, one-way ANOVA followed by a Holm-Sidak multiple comparison test was performed using GraphPad Prism (version 6.00 for Windows, GraphPad Software, La Jolla, California, USA: https://www.graphpad.com). Pearson coefficient correlation was used to correlate BCVA and the number of injections. Spearman coefficient correlations were calculated between BCVA and morphological CNV details. *P* < 0.05 was considered statistically significant.

## 3. Results

The enrolled eyes included both naïve eyes and those previously treated with anti-VEGF. The mean BCVA at baseline was 33.67 ± 15.85 ETDRS letters; at the end of the study, it was 25.27 ± 20 ETRDS letters. The difference was not statistically significant (*P* = 0.06, *r*^2^ = 0.10).

Almost half of the participants had stable BCVA, although there were some eyes in which BCVA decreased dramatically. Two possible visual acuity patterns can be observed in CNV evolution: increased or stable (positive evolution) or decreased vision (negative evolution) (Table 2).

### 3.1. Positive Evolution: Stabilization and Chronicity

**Figure 1:**
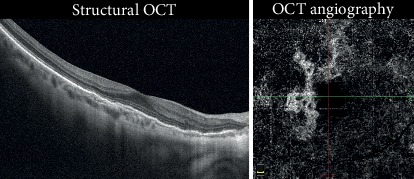
Stabilized CNV. Structural OCT shows the absence of intraretinal exudative details with an irregular profile of the RPE in the foveal region. OCTA revealed a flow signal with a well-defined outline and the absence of a dark halo. This eye received 3 intravitreal injections and became stable for more than 25 months (Case number 7).

We observed positive evolution in 55% of the patients, consisting of CNV stabilization (20%) and CNV chronicity (35%). We considered CNV stabilization as long-term remission and an absence of fluid or hemorrhaging for more than 6 months; the CNV seemed to stop developing, and no activity signals were seen. The clinical appearance showed no exudation or fluid occurrence (Figure 1). Even if there was no CNV exudation, the CNV area was larger at the end of the observation period, growing from 0.68 mm^2^ to 1.68 mm^2^.

**Figure 2:**
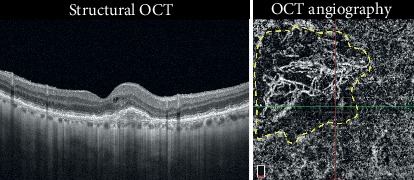
Chronic CNV. Structural OCT shows the presence of exudations with intraretinal cystic spaces. Stratified hyperreflective material below the RPE can be observed in the foveal region. OCTA revealed the presence of a flow signal with growth of thin capillary leaves and a dark halo around the CNV. The dotted yellow outline highlights the dark halo. These eyes required multiple treatments to remain stable (Case number 13).

CNV chronicity was considered when the neovascularization was consistently responsive to anti-VEGF treatment but required repetitive reinjections. In these cases, acute disease developed into chronic disease. The CNV was often quiescent with consistent and frequent recurrences (Figure 2). The CNV area was consistently larger at the end of the observation period, growing from 0.7 mm^2^ to 1.4 mm^2^.

### 3.2. Negative Evolution: Fibrosis, Atrophy, Hemorrhage, or RPE Tears

**Figure 3:**
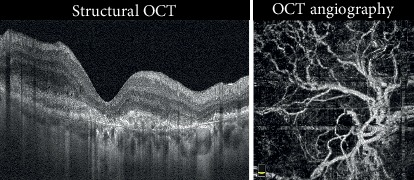
Fibrotic CNV. Structural OCT shows the presence of minimal exudation above the hyperreflective material below the RPE. Bruch's membrane is well evidenced, as well as the choroidal vessels. OCTA revealed the presence of a large flow signal with a main trunk and a “dead tree” feature. This eye had poor visual acuity, which did not improve after injection (Case number 17).

**Figure 4:**
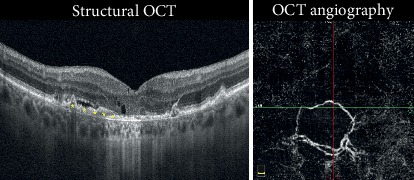
CNV associated with atrophy. Structural OCT shows minimal exudation as intraretinal cystic spaces and subretinal fluid above and hyperreflective material below the RPE (yellow asterisks). Foveal backscattering is well observable for the RPE atrophy behind the choroidal vessels. OCTA reveals the presence of a round flow signal with a growth capillary fringe. This eye required injections, but the presence of atrophy compromised visual recovery (Case number 22).

**Figure 5:**
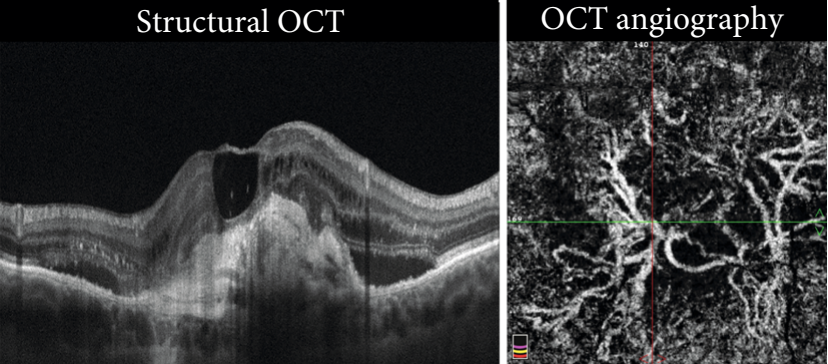
CNV with RPE tears. Structural OCT shows deconstruction of the neuroretinal tissue and large pigment epithelium detachment associated with RPE tearing. The backscattering close to the RPE indicates the tear margin. Exudation is evident, with intraretinal cystic spaces and subretinal fluid above the RPE tear. OCTA highlights the presence of the 2 main vascular trunks with multiple growth capillary fringes (Case number 28).

Negative evolution was observed in 45% of the cases, which included fibrosis (18%) (Figure 3), atrophy (25%) (Figure 4), and hemorrhage or RPE tears (2%) (Figure 5).

**Figure 6:**
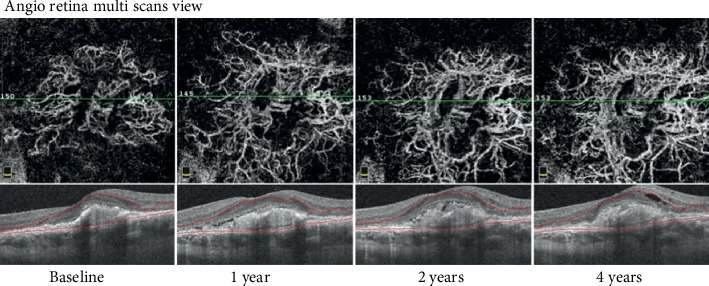
OCT angiography of type 1 CNV treated with anti-VEGF at different points in the long-term follow-up. The CNV became larger, thicker, and straighter over time. The final NV area was larger than the CNV area before treatment, and the visual acuity worsened (Case number 32).

Long-term monitoring of CNV evolution showed that the new vessels become larger, thicker, and straighter. No thin capillaries or fine loops were visible. For any evolution type, the vessel area was larger after treatment than before treatment (Figure 6).

**Figure 7:**
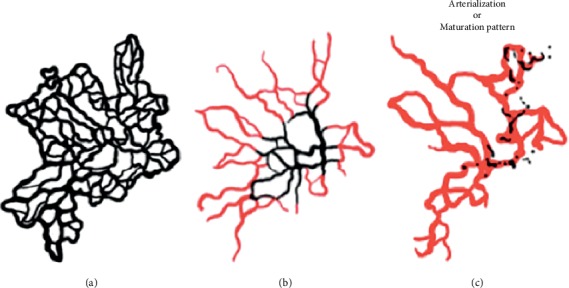
Drawing of the CNV evolution after multiple treatments. Before treatment (a), the CNV has a greater proportion of small branching vessels and peripheral arcades, indicating an active lesion. After treatment (b), the same main vessels appear to return with increased flow and decreased branch density (vessels in red). Some main branches are less affected by the treatment (vessels in black). After several anti-VEGF treatments, the CNV shows fewer complex features. This morphological pattern corresponds to the “maturation pattern” of treatment (c).

After each treatment, the same main vessels appeared to return with increased flow and decreased branch density. It appeared as though some of the main branches were less affected by the treatment. As previously described by Spaide, the onset of a complex pattern after treatment induced a less complex feature of CNV, arterialization, to become detectable [18]. We defined this morphological pattern as a “maturation pattern” (Figure 7).

**Figure 8:**
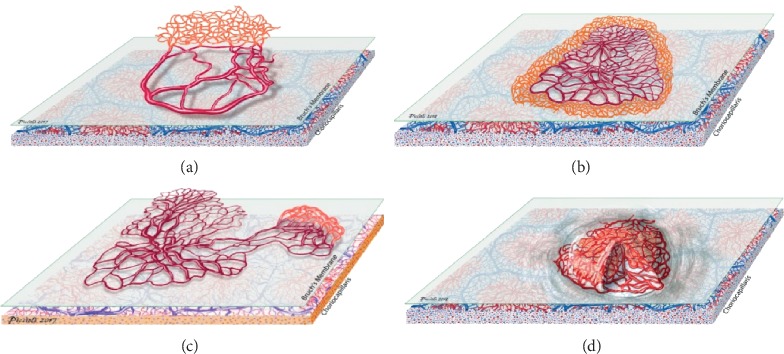
CNV growth patterns. (a) Asymmetric growth, (b) symmetric growth, (c) finger-like projections. (d) We observed a new type of CNV growth, “inside the fibrous capsule,” in which the vascular density increased instead of the vascular area.

Similarly, in agreement with the results of the study by Xu et al., we observed 3 CNV growth patterns: symmetric growth, asymmetric growth, and finger-like projections [19]. Furthermore, we observed a new entity of CNV growth, “inside the fibrous capsule.” This CNV grows in vascular density but not in area. In this specific case, the CNV grew in vascular density inside a fibrous capsule (Figure 8).

Correlation between BCVA and number of injections was not statistically significant (*P* = 0.23, *r*^2^ = 0.02) as well the correlation between CNV evolution (*P* = 0.06, *r*^2^ = −0.23) and CNV growth pattern (*P* = 0.69, *r*^2^ = −0.05).

## 4. Discussion

Although OCT angiography continues to be developed, it is useful for several visual disorder indications, particularly in the management of AMD. The analysis of neovascular flow without dye injection with OCTA allows detailed monitoring of the different CNV evolutions. Our results show that type 1 CNV has various evolution patterns, which were analyzed over a 4-year observation period.

During long-term type 1 CNV evaluation, recurrence was frequent, and we observed two dissimilar evolutions, positive evolution and negative evolution, occurring independent of the treatment [20].

Type 1 CNV “positive evolution,” manifesting as stabilization or improvement, corresponded to 20% of cases, while that manifesting as chronicity corresponded to 35% of cases. “Negative evolution” included fibrosis, which was observed in 18% of eyes, chorioretinal atrophy, which was observed in 25% of eyes, and hemorrhage or RPE tears, which was observed in 2% of eyes.

In almost all eyes, after the loading phase of the 3 intravitreal anti-VEGF injections, the disappearance of CNV ramifications but not of the main CNV trunk could be observed by OCTA. These findings suggested that it would be very difficult to predict the prognosis from OCTA findings after 3 loading doses.

Most eyes with chronic evolution had periodic reactivation after treatment, with a periodicity of 50 to 60 days after each intravitreal injection. Before the first injection and between recurrences, we observed a dark halo around the CNV of approximately 50 microns in diameter. Although the meaning of the dark halo is still controversial [21–24], in our opinion, it is due to blood sequestering by neovascularization reactivation; an increased dark halo means CNV growth [25].

The cycles seemed to be quite regular. After each treatment, the same main vessels appeared to return with increased flow and decreased branch density. The normal cyclic recurrence was extensive and generally global, although it could be localized to a segment of the CNV. In a few cases, acute nonperiodic reactivation occurred independently from treatment. This type of reactivation could take the shape of a shoot, bud, sprout, or outgrowth and may have had a specific location: terminal, axillary, lateral, fingerlike, or adventitious.

The retinal effect of repeated anti-VEGF treatments is still controversial. However, Christenbury et al. recently described that the multilayered PED aspect after chronic VEGF suppression in type 1 CNV may confer a protective effect on the overlying retinal pigment epithelium and outer retina [13].

The ability of OCTA to assess and quantify CNV may highlight activity biomarkers and guide the evaluation, treatment, and monitoring of neovascularization.

According to our previous study and to a recent observation by Al-Sheikh et al., the morphological evaluation of CNV by OCTA can distinguish nonactive CNV lesions from exudative CNV lesions [17, 20].

In contrast to the results of previous studies [7], in our study, we observed BCVA reduction at the end of follow-up. Although we were unable to determine the real reason for the BCVA decrease, we propose 3 different hypotheses: undertreatment induced by the PRN treatment, the particular aggressiveness of type 1 CNVs, and the lower starting visus compared to that in other studies.

In conclusion, type 1 CNV evolution can progress to different outcomes: stabilization, chronicity, fibrosis, atrophy, hemorrhage, or RPE tears. Approximately half of the eyes in this study followed a positive evolution, while the other half became increasingly worse. We do not know why some CNV cases became stable, with the evolution and activity signals coming to a halt; we also do not know why some CNV cases converted to chronicity. Similarly, it is unknown why some of the CNV cases had important growth, while others led to atrophy or severe fibrosis. In the future, we hope that the use of OCTA will help to better define the morphological details in the development of type 1 CNV and develop personalized medicine.

## Figures and Tables

**Table 1:** Patient demographic characteristics, baseline and final visual acuity, anti-VEGF therapy details, and follow-up interval.

Case	Age	Gender	Eye	Baseline BCVA (ETDRS)	Final BCVA (ETDRS)	Total no. of injections	Anti-VEGF agent	Follow-up (months)	CNV evolution	CNV growth pattern
1	77	F	RE	45	15	27	A	45	Atrophy	Symmetric
2	77	F	LE	30	45	28	A	36	Chronicity	Asymmetric
3	76	F	LE	35	40	29	A	58	Chronicity	Symmetric
4	78	M	RE	48	48	25	A	34	Chronicity	Asymmetric
5	78	M	LE	35	35	24	R	38	Fibrosis	Finger-like projections
6	41	F	LE	55	53	22	A	31	Chronicity	Asymmetric
7	90	F	LE	20	20	4	A	32	Stabilized	Asymmetric
8	54	F	LE	45	53	20	R	55	Chronicity	Symmetric
9	73	F	LE	40	35	14	R	40	Stabilized	Finger-like projections
10	75	F	RE	30	35	8	A	56	Stabilized	Symmetric
11	93	F	LE	48	50	7	A	38	Stabilized	Asymmetric
12	67	F	RE	50	55	3	R	36	Stabilized	Asymmetric
13	67	F	LE	20	40	8	R	36	Chronicity	Finger-like projections
14	60	M	RE	50	48	4	R	33	Stabilized	Asymmetric
15	75	M	RE	35	20	5	A	37	Stabilized	Symmetric
16	68	M	LE	55	35	12	A	54	Fibrosis	Asymmetric
17	74	F	RE	40	5	7	A	31	Atrophy	Inside fibrous capsule
18	85	M	RE	40	5	5	A	37	Atrophy	Asymmetric
19	75	F	RE	45	50	22	A	50	Chronicity	Asymmetric
20	76	F	LE	35	40	19	R	46	Chronicity	Inside fibrous capsule
21	94	F	RE	20	1	8	R	35	Hemorrage	Symmetric
22	75	M	RE	1	1	11	A	37	Atrophy	Asymmetric
23	75	M	LE	1	1	12	A	39	Atrophy	Asymmetric
24	83	F	RE	35	30	14	R	33	Chronicity	Symmetric
25	83	F	LE	35	20	11	R	33	Stabilized	Inside fibrous capsule
26	84	F	RE	45	30	12	R	33	Atrophy	Symmetric
27	87	F	RE	35	5	14	R	40	Fibrosis	Symmetric
28	87	F	LE	3	3	6	R	40	RPE tears	Finger-like projections
29	79	F	RE	50	45	9	R	38	Chronicity	Asymmetric
30	46	F	LE	20	45	11	A	46	Chronicity	Inside fibrous capsule
31	91	M	LE	20	2	14	A	32	Fibrosis	Symmetric
32	81	F	RE	5	1	13	A	33	Fibrosis	Symmetric
33	81	F	LE	50	50	14	R	33	Chronicity	Asymmetric
34	90	F	LE	5	20	12	A	39	Chronicity	Asymmetric
35	80	F	LE	48	50	14	R	58	Stabilized	Symmetric
36	72	F	LE	35	2	5	A	39	Hemorrage	Finger-like projections
37	88	M	RE	35	45	12	R	40	Chronicity	Asymmetric
38	77	F	RE	50	50	11	A	35	Chronicity	Asymmetric
39	86	F	LE	40	5	14	A	46	Fibrosis	Asymmetric
40	88	M	RE	1	1	6	A	33	Fibrosis	Asymmetric
41	95	M	LE	35	30	13	R	42	RPE tears	Symmetric
42	68	M	LE	53	53	9	R	39	Chronicity	Asymmetric
43	92	F	RE	20	5	9	R	32	Atrophy	Asymmetric
44	95	M	RE	48	30	12	A	37	Atrophy	Asymmetric
45	73	M	LE	45	45	7	A	38	Chronicity	Asymmetric
46	94	F	LE	1	1	9	R	39	Atrophy	Asymmetric
47	75	F	RE	30	30	5	R	39	Stabilized	Asymmetric
48	75	M	LE	35	20	7	A	31	Atrophy	Symmetric
49	87	F	LE	30	30	8	R	34	Chronicity	Asymmetric
50	54	F	RE	50	45	5	R	34	Fibrosis	Symmetric
51	54	F	LE	35	2	5	R	34	Fibrosis	Symmetric

RE: right eye; LE: left eye; A: aflibercept; R: ranibizumab; BCVA: best-corrected visual acuity; F: female; M: male.

**Table 2:** Type 1 CNV long-term evolution.

Evolution of disease	NV Evolution	%	TOT (%)
Positive evolution	Stabilized NV	20	55
	Chronic NV	35	

Negative evolution	Fibrosis	18	45
	Atrophy	25	
	Hemorrhage or RPE tears	2	

NV: neovascularization; RPE: retinal pigment epithelium.

## Data Availability

The data used to support the findings of this study are available from the corresponding author upon request and were included within the supplementary information file(s).

## Additional Points

*Précis*. The long-term evaluation of type 1 choroidal neovascularization (CNV) showed different outcomes for different cases: stabilization, chronicity, fibrosis, atrophy, hemorrhages, or retinal pigment epithelium (RPE) tears. We do not know why some CNV cases became stable, bringing their evolution and activity indicators to a standstill. Similarly, it is unknown why some CNV cases led to significant growth and others led to hemorrhage, atrophy, or severe fibrosis. In the future, we hope that the use of optical coherence tomography angiography (OCTA) will better define the details of the development of type 1 CNV.
